# Oral plant-derived exosome-like nanovesicles: a new therapeutic perspective for intestinal diseases

**DOI:** 10.3389/fphar.2026.1827379

**Published:** 2026-05-29

**Authors:** Lin Zhang, Muhammad Zubair, Ying Chu

**Affiliations:** 1 Central Laboratory, Wujin Hospital Affiliated with Jiangsu University, Changzhou, China; 2 Jiangsu Key Laboratory of Medical Science and Laboratory Medicine, Jiangsu University, Zhenjiang, China

**Keywords:** gastrointestinal diseases, gut microbiota, inflammatory bowel disease (IBD), oral delivery, plant-derived exosome-like nanovesicles (PELNs)

## Abstract

Plant-derived exosome-like nanovesicles (PELNs) are a class of nanoscale vesicles isolated from plant cells. They are enriched with proteins, lipids, nucleic acids and other bioactive components, and are capable of exerting diverse biological effects. Due to their efficient absorption and transformation, PELNs when administered orally, demonstrate significant therapeutic efficacy for treating various gastrointestinal diseases, including inflammatory bowel disease, intestinal tumors, and gut dysbiosis. Despite growing evidence highlighting their promise as emerging biotherapeutic agents for intestinal disorders, challenges still persist due to poorly defined mechanisms of action and limited clinical experience. This review summarizes the characteristics of PELNs, the advantages of their oral delivery, and current engineering strategies. It further explores the mechanisms through which PELNs promote intestinal health and analyzes their clinical prospects and challenges. The review aims to provide insights for future research and clinical translation, thereby facilitating the advancement of PELN-based therapies for gastrointestinal diseases.

## 1 Introduction

Extracellular vesicles (EVs) are heterogeneous, lipid bilayer-enclosed vesicles secreted by cells. Among their subtypes, exosomes (30–150 nm) are generated via multivesicular bodies and serve as key mediators of intercellular communication, playing critical roles in both physiological and pathological processes (Li et al., 2026).

In recent years, plant-derived exosome-like nanovesicles (PELNs), an emerging class of natural nanocarriers, have attracted increasing attention due to their favorable biocompatibility and diverse biological functions. Structurally similar to animal-derived exosomes, PELNs possess a lipid bilayer; however, their composition varies depending on the plant species, and they are enriched with various functional proteins, miRNAs, and endogenous bioactive compounds. They have demonstrated remarkable potential in regenerative medicine, cancer therapy, and the treatment of inflammatory diseases (Bai et al., 2025).

Advancing insights into the gut microbiome and its interaction with intestinal health have progressively shaped the evolution of therapeutic strategies for gastrointestinal diseases. In the management of these disorders, conventional pharmacological agents often face limitations including low bioavailability and systemic side effects. In contrast, PELNs, characterized by their nanoscale dimensions (30–150 nm) (Yu et al., 2025), negative surface charge, and lipid bilayer structure (Ma et al., 2025), offer distinct advantages when administered orally. They are capable of penetrating the intestinal mucus barrier and resisting extreme pH conditions and enzymatic degradation. Subsequently, they bind to intestinal epithelial cells via electrostatic interactions, thereby exhibiting notable targeting specificity and structural stability (Wang D. et al., 2024).

Accumulating evidence indicates that PELNs can effectively deliver bioactive cargo, modulate the metabolic activities of gut microbiota, influence host immune responses, and promote intestinal mucosal repair. These functions thus play a significant role in the treatment of inflammatory bowel disease (IBD) and related conditions (Tuo et al., 2025). Moreover, PELNs demonstrate the ability to selectively target intestinal tumor cells, suppress pro-inflammatory signaling, and remodel the tumor microenvironment, which offers a promising therapeutic strategy for intestinal malignancies (Li J. et al., 2025). Furthermore, engineered modifications to PELNs can substantially enhance their targeting specificity and therapeutic efficacy, opening new avenues for the treatment of gastrointestinal disorders (Ning et al., 2025).

This review examines the application of orally administered PELNs for managing gastrointestinal diseases. It also elucidates their mechanisms of action and highlights potential directions for future research.

## 2 Overview of PELNs

### 2.1 Definition of PELNs

PELNs are nano-sized vesicles isolated from plants, whose diameters typically range from 30 to 150 nm. They generally exhibit cup-shaped or saucer-shaped morphology, have a complete lipid bilayer membrane, and maintain stability in cellular environments (Sha et al., 2024). As natural drug delivery systems and therapeutic carriers, PELNs demonstrate substantial potential and have increasingly attracted research interest in the fields of nanomedicine and plant functional studies (Fan et al., 2022).

### 2.2 Biogenesis of PELNs

**FIGURE 1:**
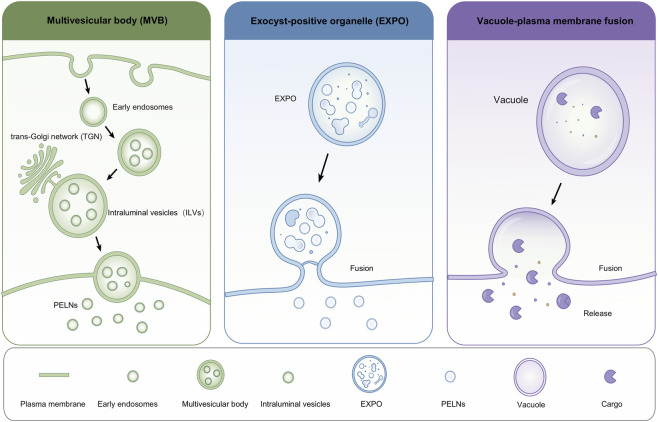
Biogenesis of PELNs. PELNs biogenesis proceeds via three primary mechanisms: the classical MVB pathway, the exocyst-positive organelle (EXPO) pathway, and the vacuole-plasma membrane fusion pathway, respectively.

Unlike animal-derived exosomes, PELNs are generated via unique biosynthetic pathways. The biogenesis of animal exosomes is well-characterized, primarily involving the double invagination of the plasma membrane and the formation of multivesicular bodies (MVB), which encompasses four stages: initiation, endocytosis, MVB formation, and secretion (van Niel et al., 2018). PELNs biogenesis primarily proceeds via three mechanisms: the canonical multivesicular body (MVB) pathway, the exocyst-positive organelle (EXPO) pathway, and the vacuole-plasma membrane fusion pathway (Song et al., 2025a) (Figure 1).

MVB pathway is the release pathway most similar to that of animal exosomes. This process begins with inward budding of the plasma membrane, leading to the formation of early endosomes (EEs). These EEs mature and interact with the trans-Golgi network, thereby facilitating the maturation of MVB (Scheuring et al., 2011). The endosomal membrane generates intraluminal vesicles (ILVs) through inward invagination. ILVs serve as key carriers selectively encapsulating bioactive molecules such as RNA, proteins, and lipids. When MVB fuse with the plasma membrane, ILVs are secreted into the extracellular space as PELNs, where they play important roles in intercellular and even inter-organismal signaling (Li et al., 2018).

EXPO was first identified in Arabidopsis and tobacco cells. Upon fusion of EXPO with the plasma membrane, it releases its internal single-membrane vesicles into the apoplastic space. This pathway is independent of the classical MVB pathway and represents a plant-specific vesicle secretion mechanism (Wang et al., 2010).

In addition to the MVB and EXPO pathways, plant vacuoles can also directly fuse with the plasma membrane to release their contents, such as hydrolytic enzymes, into the extracellular space during defense responses; this process may contribute to the biogenesis of PELNs (Shimada et al., 2018; Hu et al., 2020).

### 2.3 Composition and functions of PELNs

PELNs are enriched with diverse lipids, proteins, nucleic acids, and metabolites. These constituents act synergistically to confer a range of biological functions on plant-derived, exosome-like nanovesicles.

#### 2.3.1 Lipids

The fundamental structure of PELNs is a phospholipid bilayer composed of common phospholipids such as phosphatidic acid (PA), phosphatidylcholine (PC), phosphatidylethanolamine (PE), and plant-specific lipids including digalactosyldiacylglycerol (DGDG) and monogalactosyldiacylglycerol (MGDG) (Chen F. et al., 2025). For instance, lipidomic analysis of vesicles derived from *Arabidopsis thaliana* rosette leaves revealed a high abundance of sphingolipids, mainly glycosyl inositol phosphoceramides (GIPCs). These components contribute to strong membrane stability of the vesicles and facilitate cross-species signaling (Liu et al., 2020). Furthermore, ginger-derived nanoparticles (GDNPs), notably rich in PA, have been shown to prevent insulin resistance in mice through the restoration of Foxa2-mediated signaling homeostasis in intestinal epithelial cells (Kumar et al., 2022).

#### 2.3.2 Proteins

To date, several publications have provided data on potential protein markers of PELNs, but a consensus on their definitive identity has not yet been reached (Rodríguez de Lope et al., 2025). However, unlike animal-derived exosomes, PELNs contain aquaporins that mediate transmembrane water transport, heat shock proteins (HSPs) involved in both biotic and abiotic stress responses and in the regulation of plant growth, and annexins that mediate intracellular vesicle transport and exosome release (Di Giulio et al., 2023). For example, proteomic analysis of platycodon grandiflorus-derived exosome-like nanoparticles (PGLNs) identified 112 proteins, among which 37 were localized to the cell membrane and 15 to the plasma membrane. The Gene Ontology (GO) and the Kyoto Encyclopedia of Genes and Genomes (KEGG) pathway analyses indicated their involvement in signal transduction and multiple metabolic pathways (Fu et al., 2025). These proteins not only participate in vesicle formation and release, but also play critical roles in receptor recognition, cell adhesion, and immunomodulation.

#### 2.3.3 Nucleic acids

Regarding their nucleic acid cargo, PELNs contain various small RNAs such as miRNAs and siRNAs, as well as mRNAs, tRNAs, and lncRNAs (Ambrosone et al., 2023). Among these, miRNAs can regulate target gene expression and mediate cross-kingdom metabolic regulation (Urzì et al., 2021). For example, the core miRNA effectors aof-miR168a and osa-miR164a in polygoni multiflori radix-derived exosome-like nanoparticles (PMENs) directly target the human androgen receptor (AR) via conserved 3′UTR binding sites, which inhibits AR expression and offers a promising strategy for treating androgen-driven disorders (Li S. et al., 2025). Lycium ruthenicum murray-derived exosome-like nanoparticles (LRM-ELNs) can deliver ata-miR156c3p to mitigate neurodegenerative pathology through the MAPK and PI3K/AKT signaling pathways (Zhang et al., 2024). Similarly, ginger-derived exosome-like nanoparticles (GELNs) carry aly-miR159a-3p, which enhances anti-PD-L1 therapeutic efficacy in melanoma (Teng et al., 2025). The therapeutic activities of PELNs-derived miRNAs are summarized in the table below (Table 1).

**TABLE 1:** miRNAs packaged in PELNs and their therapeutic activities.

Plant source	miRNA	Therapeutic activities	Ref
Phellinus linteus	miR-CM1	Inhibit ultraviolet-induced skin aging	Han et al. (2022)
Ginseng	mtr-miR159a	Stimulate neural differentiation of stem cells	Xu et al. (2021)
Lyciumruthenicum murray	ata-miR156c3p	Alleviate neurodegenerative diseases	Zhang et al. (2024)
Ficus carica	peu-miR-2916-p3	Inhibit bone metastasis of breast cancer	Wang et al. (2026a)
Ginger	mdo-miR7267–3p	Shape the gut microbiota	Teng et al. (2018)
Ginger	aly-miR159a-3p	Enhance anti-PD-L1 efficacy	Teng et al. (2025)
Polygoni multiflori radix	aof-miR168a and osa-miR164a	Promote hair growth	Li et al. (2025b)
H. cordata	miR858a and miR858b	Against respiratory RNA viruses	Zhu et al. (2023a)
Brucea javanica	B-E-miRs	Inhibit breast cancer	Yan et al. (2024a)

#### 2.3.4 Other metabolites

In addition, PELNs contain secondary metabolites including flavonoids, naringin, and glucosinolates, which confer diverse biological activities such as antioxidant, anti-inflammatory, and antimicrobial properties (Ambrosone et al., 2023). Exosome-like nanoparticles derived from citrus reticulata blanco (CEVs) contain three citrus flavonoids, namely, sinensetin, naringin, and neohesperidin, and these bioactive components confer potent antioxidant and anti-inflammatory activities to CEVs (Li et al., 2023). Zhou et al. identified a total of 564 metabolites in goji-derived exosomes (GqDNVs), among which the five most abundant classes were free fatty acids (11%), amino acids and their derivatives (10%), phenolic acids (8%), saccharides (7%), and alkaloids (7%) (Zhou et al., 2025). Kim et al. identified 17 major metabolites in aster yomena callus-derived exosome-like nanoparticles (AYC-EVs) and demonstrated their immunomodulatory potential in alleviating allergic asthma (Kim et al., 2022).

Recent studies have demonstrated that PELNs not only are involved in plant growth, development, and pathogen defense, but also modulate physiological processes in animal cells, highlighting their broad potential for interdisciplinary applications in the fields of medicine, nutrition, and beyond (Shalvina et al., 2026).

### 2.4 Extraction and purification of PELNs

**FIGURE 2:**
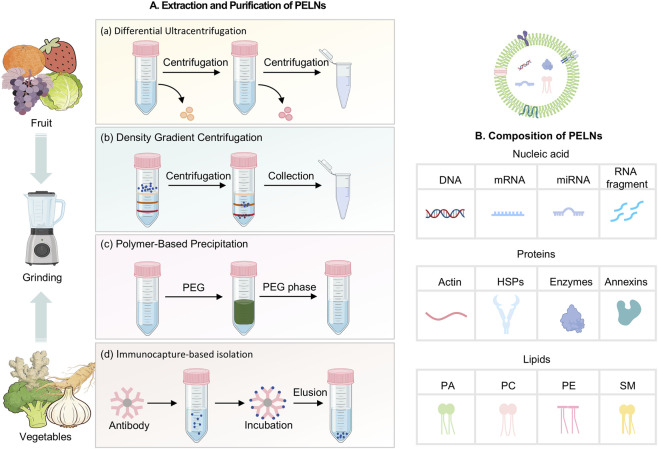
Extraction methods and composition of PELNs. A. Extraction and purification of PELNs. **(a)** Differential ultracentrifugation **(b)** Density gradient centrifugation **(c)** Polymer-based precipitation **(d)** Immunocapture-based isolation. B. Composition of PELNs. Consisting of nucleic acids, proteins, lipids and other substances.

Extraction and purification of PELNs are fundamental steps for elucidating their biological functions. To obtain high yields of exosome-like vesicles, fresh and succulent plant tissues, such as leaves or fruits are preferred as starting materials (Figure 2). The advantages and disadvantages of currently commonly used isolation techniques for PELNs are shown in Table 2.

**TABLE 2:** Methods for isolation of PELNs.

Methods	Principle	Advantages	Disadvantages	Ref
Differential ultracentrifugation	Removes cellular debris via sequential low-speed spins; pellets exosomes at 100,000 × g	Simple and widely applicable	High cost, time-consuming, potential structural damage	Chen et al. (2019), Luo et al. (2025)
Density gradient centrifugation	Sucrose density gradient separates PELNs based on buoyant density	Enables high-purity PELN isolation	Requires gradient optimization	Chen et al. (2026), Xiao et al. (2025a)
Size exclusion chromatography	Separates vesicles based on differences in hydrodynamic radius	Preserves structural integrity and bioactivity	Not suitable for large-scale production	Li et al. (2024a), You et al. (2021)
Polymer-based precipitation	Addition of PEG induces co-precipitation of PELNs	Simple to operate	Low purity	Kim et al. (2023a), Ming et al. (2025), Chen et al. (2025b), Madhan et al. (2024)
Affinity chromatography	Specifically recognizes membrane surface markers	Improves capture efficiency and purity	High cost, demanding storage conditions	Huang et al. (2021)

#### 2.4.1 Differential ultracentrifugation

Differential ultracentrifugation involves low-temperature grinding or pressing of plant tissues to extract sap, followed by filtration through a mesh filter to remove large particulate matter. Different centrifugal speeds are then applied sequentially to remove impurities such as large cell debris, and ultimately a centrifugal force of 100,000 × g is used to achieve effective separation of exosomes from soluble proteins and other impurities (Chen et al., 2019). This method is the most widely applied and is suitable for various plant samples. However, it requires expensive equipment, is time-consuming, may compromise vesicle integrity, and reduces the yield of functional PELNs. In the context of inflammatory bowel disease therapy, achieving therapeutic efficacy requires sufficient doses of PELNs to reach the lesion site; however, excessive centrifugal force may induce structural damage to exosomes. Therefore, to meet clinical therapeutic demands, the centrifugation process requires further optimization, potentially in combination with other methods, to enhance exosome yield and stability. To this end, scientists have explored a novel approach——tangential flow filtration (TFF). Compared to ultracentrifugation, the use of a filter membrane with a molecular weight cut-off (MWCO) of 750 kDa, combined with the maintenance of transmembrane pressure (TMP) between 3 and 5 psi, resulted in a threefold increase in the yield of EVs (Luo et al., 2025).

#### 2.4.2 Density gradient centrifugation

Density gradient centrifugation is a technique that utilizes a discontinuous or continuous gradient of sucrose solutions, onto which crude exosomes obtained by differential centrifugation are layered. Subsequently, ultracentrifugation is performed to facilitate the accumulation of PELNs within distinct density fractions (Chen et al., 2026). For fiber-rich plant tissues, pretreatment with 30 mg/mL cellulase and 2 mg/mL pectinase for 12 h is employed to degrade the plant cell wall and promote the release of PELNs (Xiao Y. et al., 2025). The advantages of this method include the high purity of the isolated PELNs and suitability for large-scale production.

#### 2.4.3 Size exclusion chromatography

Size exclusion chromatography is a fractionation method that separates vesicles according to differences in their hydrodynamic radius. This gentle separation process involves no shear forces and effectively preserves the native morphology and functional activity of exosomes (Li Y. et al., 2024). A study reported that the purity of cabbage-derived exosomes isolated by size exclusion chromatography was 10 × 10^9^ particles μg^−1^ protein, whereas those obtained by ultracentrifugation and PEG precipitation were 0.432 × 10^9^ and 0.242 × 10^9^ particles μg^−1^ protein (You et al., 2021).

#### 2.4.4 Polymer-based precipitation

Polymer precipitation involves adding polymers such as polyethylene glycol to modify the solubility properties of the medium, leading to co-precipitation of PELNs. The concentration of PEG solution typically ranges from 15% to 20%. For example, filtered balloon flower root juice was combined with 16% PEG solution and incubated for 16 h, followed by centrifugation to pellet the precipitate, yielding balloon flower root-derived exosomes (BFR-EVs) (Kim M. et al., 2023). Ginger-derived exosome-like nanoparticles (GELNs) were precipitated using 20% PEG solution (Ming et al., 2025). Li et al. developed an oral drug delivery system utilizing mulberry leaf-derived exosome-like nanoparticles (SN-MNs), which were subsequently modified with DSPE-PEG2000 to improve their intestinal stability (Chen L. et al., 2025). While this method is operationally simple and requires no specialized equipment, it typically results in relatively low product purity (Madhan et al., 2024).

#### 2.4.5 Emerging methods

In recent years, with the advancement of nanomaterials and surface modification technologies, a series of novel separation strategies have emerged. For instance, methods based on functionalized magnetic nanoparticles or affinity chromatography can selectively target membrane surface markers, significantly enhancing the capture efficiency and purity of exosomes (Huang et al., 2021). These new material-assisted extraction strategies demonstrate superior recovery rate and operational reproducibility. However, our understanding of the surface composition of PELNs remains limited, and the potential of immunoaffinity-based methods for PELNs extraction remains underexplored.

Nevertheless, it is important to recognize that plant-derived impurities such as fiber debris may trigger adverse immune responses or interfere with intestinal barrier penetration. The PELNs obtained by the currently widespread disruptive isolation methods are, in fact, a mixture containing intracellular fragments and artificial vesicles. Therefore, it is imperative to establish more standardized molecular markers for plant EVs and to seek optimized protocols to obtain “genuine extracellular vesicles” (Bifolco et al., 2026).

## 3 Oral PELN-based therapeutics

Although diverse strategies for the extraction and purification of PELNs have been developed, the ultimate purpose of these techniques is ultimately to facilitate drug delivery. Among the numerous administration routes, oral delivery offers unique advantages due to its non-invasive nature and distinct advantage for intestinal diseases. Consequently, further exploration of the therapeutic potential of PELNs for oral drug delivery is warranted.

### 3.1 Advantages of orally administered PELNs

Oral administration is a common method of drug delivery, widely adopted in clinical practice because it is highly convenient and relatively low in cost (Lombardi et al., 2019). Compared to alternative delivery approaches such as injections, oral administration offers distinct advantages including non-invasiveness, ease of self-administration, and lower overall cost (Caffarel-Salvador et al., 2017).

In recent years, PELNs have attracted considerable interest as a promising class of oral delivery vehicles. The structural stability of PELNs in the gastrointestinal environment underpins their utility as oral carriers. Studies have demonstrated that PELNs retain their physicochemical properties, such as particle size and zeta potential, under simulated gastric conditions, while preserving the integrity of internal bioactive components, including proteins, RNAs, and small molecule compounds. These observations indicate that PELNs have the ability to withstand extreme pH fluctuations and enzymatic degradation (Teng et al., 2018). Upon oral ingestion, PELNs can deliver encapsulated bioactive cargo precisely to target regions within the gastrointestinal tract (Yang et al., 2020; Zhang et al., 2016a). Owing to this capability, PELNs are considered as naturally derived oral delivery platform with significant potential (Dad et al., 2021). This property not only enhances the bioavailability of therapeutic agents but also may reduce systemic side effects associated with drug exposure, thereby underscoring the unique value of PELNs in the targeted treatment of gastrointestinal disorders (Zuo et al., 2025).

Compared to intravenously administered exosomes, orally delivered PELNs demonstrate distinct therapeutic advantages in terms of efficacy and safety. As reported by Chae et al., intravenously injected exosomes predominantly accumulate in the liver and spleen, with minimal distribution to the intestinal tract (Hong et al., 2021). In contrast, oral administration of these exosomes exhibits superior efficacy in alleviating colitis compared to intravenous administration. Furthermore, Chen et al. observed that orally administered tea flower-derived exosome-like nanovesicles (TFENs) display low toxicity and high biocompatibility, whereas intravenous injection of TFENs increases metabolic burden, triggers immune activation, induces nephrotoxicity, and alters hematological parameters (Chen et al., 2022). Thus, the oral route for PELNs not only facilitates targeted drug release but also offers a novel interventional strategy for the management of gastrointestinal diseases.

### 3.2 Absorption and translocation of PELNs

The bioavailability of PELNs largely depends on their absorption mechanisms and biotransformation processes *in vivo*. Studies have shown that the intestinal mucus layer is primarily composed of MUC2 mucin, with pore sizes ranging from approximately 20–200 nm (Round et al., 2012). Under this physical barrier, PELNs with small particle sizes exhibit enhanced diffusivity through the mucus layer and reach the surface of intestinal epithelial cells (Ning et al., 2025). The surface lipid composition of PELNs may also influence their intestinal absorption. One study demonstrated that Catharanthus roseus-derived exosome-like nanovesicles (CLDENs) contain 36.5% ether-phospholipids. Since ether-phospholipids increase the rigidity and chemical stability of the lipid bilayer, this unique lipid composition may enhance their diffusion behavior in mucus (Ou et al., 2023).

Subsequently, intestinal epithelial cells internalize PELNs through endocytosis, and their cargo may be subsequently released into the cytoplasm (Kim J. et al., 2023). Yin et al. isolated ginger-derived exosome-like nanoparticles (GELNs) characterized by a particle size of 156 ± 36 nm and a zeta potential of −26.6 ± 5 mV, and demonstrated that GELNs are specifically internalized by intestinal cells through caveolin-mediated endocytosis and macropinocytosis (Yin et al., 2022).

In addition to epithelial cells, macrophages in the intestinal lamina propria can facilitate the uptake of PELNs through phagocytosis, thereby influencing their systemic distribution (Mu et al., 2014). This mechanism is further corroborated by bioengineering studies. Yang et al. fabricated resveratrol nanocrystals (RNs) and subsequently encapsulated them within FP127-functionalized mulberry leaf nanoparticles (MLNs). After oral administration, FP127@RN-MLNs remained stable during transit through the gastrointestinal tract and were specifically released into the colonic lumen, ultimately undergoing internalization by colonic epithelial cells and macrophages (Yang et al., 2023).

Furthermore, orally administered PELNs interact with the gut microbiota. Wang et al. found that pueraria lobata-derived exosome-like nanoparticles (Pu-ELNs) were preferentially taken up by the commensal gut bacterium Ruminococcus gnavus. Via their lipid components, Pu-ELNs delivered gma-miR4412 into R. gnavus, which directly inhibited the expression of its phenylalanine decarboxylase, thereby reducing the production of the bacterial metabolite phenylethylamine (PEA) (Han et al., 2025). Cistanche deserticola-derived exosome-like nanovesicles (CELNs) increased the abundance of GABA-producing bacterial genera (such as *Lactobacillus* and *Bacteroides*), elevated intestinal GABA levels, and subsequently activated GABAA receptor subunits α2 and β2/3, a process potentially involving the absorption and transformation of CELNs (Zhang et al., 2025). These results indicate that PELNs interact with gut microbiota, thereby modulating the metabolic fate of these nanoparticles.

Overall, the *in vivo* absorption and transport of PELNs are coordinately regulated by multiple key factors, including particle size, surface charge, lipid composition, and the intestinal microbial environment, which collectively determine their mucus diffusion capacity, cellular uptake efficiency, and *in vivo* distribution characteristics. Following intestinal absorption, PELNs can enter systemic circulation and distribute to various organs, where their subsequent biodistribution is modulated by particle size, surface charge, and plasma protein adsorption. Studies have shown that nanoparticles exhibit time-dependent distribution patterns *in vivo*, achieving peak tissue concentrations within hours after administration, followed by gradual clearance (Zhu M. Z. et al., 2023).

## 4 Engineering of PELNs

Conventional drug delivery systems face several limitations. Many therapeutic agents are inherently unstable *in vivo* (Queiroz et al., 2020), and are prone to degradation. Additionally, they often struggle to cross biological barriers such as the intestinal epithelium or the blood-brain barrier. Consequently, drugs may fail to accumulate specifically at the site of pathology (Tang et al., 2025).

Recent studies have demonstrated that plant-derived microscale capsules, such as pollen exine capsules, facilitate pH-responsive release of hydrophobic nutrients when coated with calcium alginate, thereby shielding the cargo from gastric degradation and facilitating intestinal delivery (Wu et al., 2018). Drawing inspiration from the design principles of these microscale delivery systems, PELNs have emerged as a class of natural nanocarriers with intrinsic pH-responsive properties. The stability of PELNs is commonly evaluated using zeta potential and particle size measurements. For instance, research has demonstrated that exosome-like nanoparticles extracted from rose (CLDENs) remained stable for at least 12 h in simulated gastric fluid and simulated intestinal fluid, exhibiting no significant alterations in particle size or zeta potential (Ou et al., 2023) To evaluate the stability of Andrographis paniculata-derived exosome-like nanoparticles (APELNs) in the gastrointestinal tract, they were incubated in simulated gastric and intestinal fluids. Compared with the PBS control group, the particle size of APELNs showed no significant changes in either the gastric or intestinal environment, indicating their resistance to both acidic and alkaline conditions. Furthermore, the fluorescence intensity of IRDye® 800CW-labeled APELNs remained stable following a 4-h incubation in simulated gastric and intestinal fluids (Zhu M. et al., 2025). These findings confirm that PELNs can remain stable in harsh gastrointestinal environments, making them promising carriers for drug delivery.

The drug loading strategies for PELNs are categorized as endogenous loading and exogenous loading. Endogenous loading refers to the integration of bioactive components into vesicles during their biogenesis, whereas exogenous loading involves the introduction of therapeutic molecules into vesicles after their isolation (Rajasekaran et al., 2025). Common exogenous loading methods include physicochemical approaches such as electroporation, sonication, freeze-thawing, and co-incubation, which facilitate the efficient encapsulation of small molecule drugs, proteins, or nucleic acids into the vesicles without compromising vesicle integrity (Xi et al., 2021; Mukhopadhya et al., 2023; Chen et al., 2024). Studies have demonstrated that these methods maintain the native size, morphology, and surface-marker profiles of the vesicles while achieving high loading efficiencies (Villata et al., 2020). As a result, researchers have successfully encapsulated drugs, miRNAs, and other therapeutic agents into PELNs, thereby substantially improving the efficacy of treatments for gastrointestinal disorders. Key strategies for drug loading into PELNs are summarized in Table 3 below.

**TABLE 3:** Drug-loading strategies for PELNs.

Plant source	Loaded cargo	Disease	Mechanism	Ref
Tea	Polydeoxyribonucleotide (PDRN)	Colitis	Activates the cAMP/HIF-1α pathway to drive M2 macrophage polarization and restore gut microbiota homeostasis	Cao et al. (2025)
Ginger	Curcumin (CUR)	Ulcerative colitis	Enhances solubility and stability of CUR in colonic fluid, improves colon length, and reduces pathological damage	Huang et al. (2024)
Ginger	Doxorubicin (Dox)	Colon tumor	Targeted delivery to the tumor site and induction of apoptosis	Zhang et al. (2016b)
Tartary buckwheat	Chlorogenic Acid (CGA) and Selenium Nanoparticles (SeNPs)	Ulcerative colitis	Increases gastrointestinal retention of CGA and SeNPs, alleviates intestinal barrier dysfunction	Li et al. (2025c)
Broccoli	miR166b-3p	Colon cancer	Protects miRNA from gastrointestinal digestion	Del Pozo-Acebo et al. (2022)
*Centella asiatica*	aof-miR396b and fvemiR396c-3p	Colitis	Attenuates inflammatory responses in the intestinal microenvironment and enhances cellular immune function	Shi et al. (2025)
Ginger	osa-miR164d	Colon tumor	Downregulates NF-κB expression, modulates macrophage polarization, and reprograms macrophage function	Yan et al. (2024b)
Coptis chinensis	miR-5106	Inflammatory bowel disease	Downregulates Slc39a2 expression and restores zinc homeostasis in neutrophils	Yang et al. (2025a)

Polydeoxyribonucleotide (PDRN) is a bioactive agent with anti-inflammatory and tissue repair properties; however, its clinical translation has been impeded by challenges related to oral delivery. Cao et al. encapsulated PDRN within tea-derived extracellular vesicles (EVs) to develop PDRN-EV nanoparticles. Experimental evidence demonstrated that PDRN-EVs accumulate in inflamed colonic tissues, activate the cAMP/HIF-1α signaling pathway, thereby promoting M2 macrophage polarization, and restore intestinal microbiota homeostasis (Cao et al., 2025).

Curcumin (CUR), a lipophilic natural polyphenol, with anti-inflammatory and antioxidant activities, faces clinical limitations due to its physiological instability and difficulty in maintaining effective concentrations at injured sites. Importantly, researchers isolated nanoparticles from fresh ginger and employed them as a safe and sustainable delivery platform for CUR. This approach increased the solubility and stability of CUR in colonic fluid, increased colon length, reduced pathological colonic damage, and alleviated ulcerative colitis (Huang et al., 2024).

In another study, nanoparticles isolated from ginger were reassembled into ginger-derived nanovectors (GDNVs), efficiently taken up by colon cancer cells. The authors further demonstrated that GDNVs could serve as a delivery platform for doxorubicin (Dox), exhibiting a superior pH-dependent drug release profile compared to commercial liposomal Dox, thereby enabling targeted delivery to colon tumor sites (Zhang et al., 2016b).

Similarly, tartary buckwheat-derived exosome-like nanoparticles (TB-ELNs) were used as carriers, with chlorogenic acid (CGA) loaded into their inner cavity and selenium nanoparticles (SeNPs) conjugated onto the outer lipid membrane, thereby constructing a dual carrier system (SeNPs ELNs CGA). Moreover, *in vitro* and *in vivo* experiments confirmed that this dual carrier design significantly enhanced the gastrointestinal retention of both CGA and SeNPs, alleviated intestinal barrier dysfunction, and did not induce hematotoxicity or hepatorenal injury upon long-term administration (Li D. et al., 2025).

MicroRNAs (miRNAs) are a class of small non-coding RNAs that regulate gene expression (Billi et al., 2024). Their broad biological activities have advanced our understanding of disease mechanisms and highlighted potential therapeutic applications. However, unprotected miRNAs are prone to rapid degradation, which necessitates developing suitable delivery strategies to enhance their therapeutic potential (Rupaimoole et al., 2011). Lorena et al. reported that loading exogenous miR-166b-3p into EVs isolated from broccoli significantly improved the survival of methylated miRNA compared to its free form, demonstrating the protective capacity of plant-derived EVs for nucleic acid cargo (Del Pozo-Acebo et al., 2022). Similarly, exosomes derived from *Centella asiatica*, enriched with aof-miR396b and fvemiR396c-3p, were shown to attenuate inflammatory responses in the intestinal microenvironment and enhance cellular immune functions (Shi et al., 2025). In another study, Yan et al. employed a microfluidic mixing device to prepare ginger-derived exosome-like nanoparticles (GELNs) loaded with osa-miR164d. The authors demonstrated that osa-miR164d directly targets the 3′-UTR of TAB1, downregulates NF-κB expression, and reprograms macrophage polarization, thereby effectively alleviating colitis-associated symptoms (Yan L. et al., 2024). Furthermore, extracellular vesicle-like nanoparticles (Cc-ELNs) from coptis chinensis were shown to deliver miR-5106, which downregulates Slc39a2 expression and restores zinc homeostasis in neutrophils, confirming Cc-ELNs as a natural and effective therapeutic agent for inflammatory bowel disease (Yang Y. et al., 2025).

In summary, cargo can be efficiently loaded into PELNs through methods such as electroporation, sonication, freeze-thawing, and co-incubation. Although PELNs hold great promise as oral drug delivery vehicles, their current drug loading methodologies still face significant technical challenges. Lipophilic substances such as curcumin are readily incorporated into vesicles, reaching up to 10^7^ molecules per vesicle without apparent saturation; in contrast, hydrophilic molecules are limited by membrane permeability, achieving saturation at approximately 10^4^ molecules per vesicle (Tréton et al., 2023). To overcome these bottlenecks, recent studies have explored novel drug loading strategies. For instance, by leveraging the intrinsic photosensitivity of Pueraria lobata-derived exosomes (PDEVs), researchers have induced ROS production via LED irradiation to transiently increase membrane permeability. The optimal PDEV-to-cargo ratio was determined to be 1:30, achieving a peak loading efficiency of 80% after 10 min of irradiation (Lu et al., 2025). This strategy offers a promising strategy for efficient loading of hydrophilic drugs, although the potential risk of oxidative damage warrants further investigation.

## 5 Clinical applications of PELNs in gastrointestinal diseases

By employing these engineering strategies, drug-loaded PELNs exhibit enhanced targeting ability and biostability, thereby improving their delivery efficiency. Leveraging these properties, PELNs have demonstrated potential therapeutic effects in various disease models, particularly showing particular promise for treating intestinal-related diseases. We will systematically summarize the research progress of PELNs in intestinal diseases categorized by their distinct mechanisms of action.

### 5.1 Immunomodulatory and anti-inflammatory effects

Inflammatory bowel disease (IBD) is a heterogeneous group of chronic intestinal inflammatory disorders, primarily driven by a disruption in host immune homeostasis. It is primarily divided into ulcerative colitis and Crohn’s disease, and there is still no definitive cure in current clinical practice (Glassner et al., 2020). Patients typically present with persistent inflammatory infiltration and tissue damage in the intestinal mucosa, characterized by a relapsing and chronic clinical course (Luo et al., 2022). The pathogenesis of IBD is strongly associated with aberrant activation of immune cells. Moreover, disruption of the intestinal barrier facilitates the translocation of harmful luminal components into the systemic circulation, thereby exacerbating systemic inflammatory responses (Ciccia et al., 2017). Understanding these pathophysiological mechanisms is crucial, therefore, for developing novel therapeutic strategies.

Macrophages play a pivotal role in innate immunity through multiple mechanisms, including phagocytosis, antigen presentation, cytokine secretion, and tissue repair, to maintain immune homeostasis. Dysregulation of the balance between M1 and M2 macrophage phenotypes can affect tissue outcomes during inflammation or injury, thereby driving the onset and progression of inflammatory damage (Wang T. et al., 2026). The shift from M2 to M1-polarized macrophages results in a sustained increase in pro-inflammatory cytokines, including TNF-α, IL-6, and IL-1β (Chang, 2020; Morales and Xue, 2023). Moreover, the excessive secretion of IL-17 and IL-23 by Th17 cells promotes chronic inflammation and concurrently suppresses the regulatory functions of Treg cells, impairing their ability to restrain immune activation (Gao H. et al., 2025).

**FIGURE 3:**
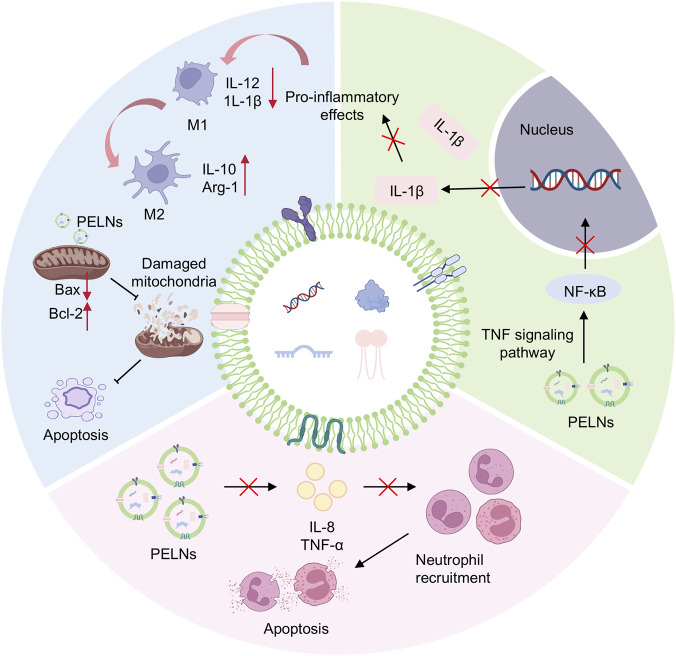
Immunomodulatory effects of PELNs. PELNs can disrupt the dynamic balance between pro-inflammatory and anti-inflammatory factors, promote macrophage polarization, upregulate the expression of IL-10 and Arg-1, and downregulate the secretion of IL-1β and TNF-α. Meanwhile, PELNs can reduce neutrophil recruitment and inhibit the formation of neutrophil extracellular traps (NETs).

Research demonstrates that PELNs modulate immune responses through multiple pathways, influencing the dynamic equilibrium between pro-inflammatory and anti-inflammatory cytokines, thereby attenuating the progression of IBD (Wu et al., 2026) (Figure 3). In a dextran sulfate sodium (DSS)-induced mouse colitis model, exosomes from red cabbage were shown to activate the PPAR-γ pathway, upregulate M2-associated markers (e.g., Arg1, IL10), and simultaneously suppress the expression of M1-related molecules such as TNFα and IL-1β (Ka et al., 2024).

Furthermore, Lycium barbarum lipid-based edible nanoparticles (LBLNs) are efficiently internalized by macrophages in ulcerative colitis. They significantly enhance the expression of the anti-inflammatory cytokine IL-10 while reducing the secretion of pro-inflammatory factors TNF-α and IL-12. *In vivo* studies have confirmed that orally administered LBLNs specifically accumulate in inflamed colon tissue, alleviating colitis-associated symptoms including colon shortening, splenomegaly, histopathological damage, and ulceration (Zu et al., 2020). Similarly, Momordica charantia-derived extracellular vesicles (MCEVs) suppress macrophage-driven inflammation, scavenge reactive oxygen species (ROS), and protect cells from oxidative injury (Gao B. et al., 2025). Liu et al. reported that orally administered curcumin-derived nanoparticles ameliorate murine colitis by modulating the expression of pro-inflammatory cytokines (TNF-α, IL-6, IL-1β) and antioxidant-related genes. The observed inactivation of the NF-κB pathway likely contributes to the resolution of colitis (Liu et al., 2022).

Neutrophil extracellular traps (NETs) physically disrupt intestinal epithelial tight junctions and induce epithelial apoptosis, serving as key drivers of exacerbation of inflammation (Leppkes et al., 2022; Long et al., 2024). Additionally, neutrophils release reactive oxygen species (ROS), myeloperoxidase (MPO), and pro-inflammatory cytokines, which directly damage the intestinal epithelium, amplify inflammatory signaling, and lead to persistent mucosal injury (Kałużna et al., 2022). Coptis chinensis-derived exosome-like nanoparticles (Cc-ELNs) have been shown to reduce neutrophil recruitment, inhibit NETs formation, and attenuate pyroptosis in intestinal epithelial cells, thereby significantly alleviating colitis (Yang Y. et al., 2025). Similarly, prunus mume-derived extracellular vesicle-like particles (PM-EVLPs) isolated using high-speed centrifugation combined with density gradient purification ameliorated colitis in mice, as evidenced by less body weight loss, reduced MPO activity, and reduced neutrophil infiltration in the colonic lamina propria (Lv et al., 2025).

Further studies indicate that purslane-derived exosomes (PELNs) promote the elevation of indole derivatives, activate the aryl hydrocarbon receptor in conventional CD4^+^ T cells, reprogram these cells into double-positive CD4^+^CD8^+^ T cells, and lower the expression of pro-inflammatory cytokines, collectively contributing to the alleviation of ulcerative colitis (Zhu M. Z. et al., 2023). Moreover, exosomes isolated from other medicinal plants such as *Zanthoxylum bungeanum* exhibit comparable anti-inflammatory effects (Gong et al., 2025).

### 5.2 Promotion of intestinal repair and regeneration

The intestinal barrier constitutes a dynamic, multi-layered defense system primarily composed of the mucus layer, epithelial barrier, and gut-vascular barrier (Pellegrini et al., 2023). Intestinal epithelial cells form a tightly sealed barrier through tight junction proteins, allowing selective nutrient absorption while preventing the penetration of bacteria, toxins, and other harmful substances into the systemic circulation. They thus serve as the first physical barrier against pathogen invasion (Sli et al., 2020; Lezutekong et al., 2018). The integrity of the intestinal barrier relies on the coordinated function of the physical, the chemical, the immune, the biological, and the neurovascular components (Takiishi et al., 2017). When impaired, bacterial toxins and other luminal agents can translocate into the bloodstream, triggering systemic inflammation and potentially contributing to central nervous system disorders.

In clinical research, intestinal permeability is commonly assessed by measuring FITC dextran flux, serum D-lactate levels, and diamine oxidase (DAO) activity, and the expression of tight junction regulatory proteins, such as occludin, serves as an indicator of intestinal barrier damage (Zhao et al., 2011).

**FIGURE 4:**
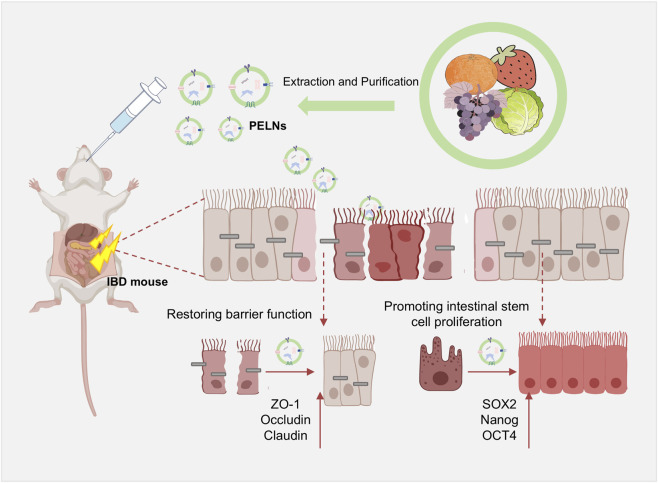
Repair effect of PELNs on the intestinal barrier. The repair effect of PELNs on the intestinal barrier is manifested by promoting the expression of tight junction proteins (ZO-1, Occludin, and Claudin) and enhancing the proliferation of intestinal stem cell-related genes, including SOX2, Nanog, and OCT4.

Recent studies have highlighted the prominent role of PELNs in the restoration of intestinal barrier integrity (Figure 4). For instance, grape-derived exosomes have been shown to penetrate the intestinal mucous barrier and be taken up by mouse intestinal stem cells. By activating the Wnt/β-catenin signaling pathway, these exosomes upregulate multiple genes that promote intestinal stem cell growth, such as SOX2, Nanog, and OCT4, thereby promoting stem cell proliferation and enhancing mucosal healing (Ju et al., 2013). Aloe-derived nanovesicles (ANVs) were found to restore the expression of tight junction (TJ) and adherens junction (AJ) proteins, thereby preventing increased intestinal permeability induced by acute colitis. This effect promoted intestinal repair and alleviated colonic inflammation (Choi et al., 2023). Ginseng-derived exosomes (GEs) suppressed the LPS-induced increase in FITC dextran flux across Caco-2 monolayers, indicating their ability to reduce intestinal epithelial permeability and repair intercellular tight junctions. Furthermore, GEs attenuated the leakage of diamine oxidase (DAO) from the intestinal mucosa into the bloodstream, consistent with reduced mucosal damage (Yang S. et al., 2025). Tea-derived extracellular vesicles (EVs) significantly restored the number of goblet cells and mucus secretion in DSS-induced colitis mice, while markedly upregulating the expression of tight junction proteins ZO-1 and Occludin (Cao et al., 2025). Similarly, citrus-derived exosomes upregulate the expression of Occludin, Claudin 1, and other TJ proteins, reducing intestinal epithelial apoptosis and promoting barrier repair (Bruno et al., 2021).

### 5.3 Anti-tumor activity

Colorectal cancer, in particular, has emerged as one of the leading causes of cancer-related mortality worldwide. In recent years, the global incidence of colorectal cancer has risen rapidly, especially in some developing regions where early screening and timely intervention remain challenging (Liu et al., 2025). Due to the challenges of early diagnosis, the majority of patients are already at an intermediate or advanced stage when diagnosed, often missing the window for surgical intervention. Moreover, more than 50% of patients develop multidrug resistance, leading to therapeutic failure. Conventional drugs are prone to degradation in the intestinal environment and lack tumor-specific targeting capability (Nezhad Mohseni et al., 2022). Exosomes, serving as key messengers within the tumor microenvironment, can not only transfer oncogenic factors to accelerate metastasis, but also act as carriers of drug resistance (Feng et al., 2022), thereby positioning them as promising targets for novel interventional strategies.

**FIGURE 5:**
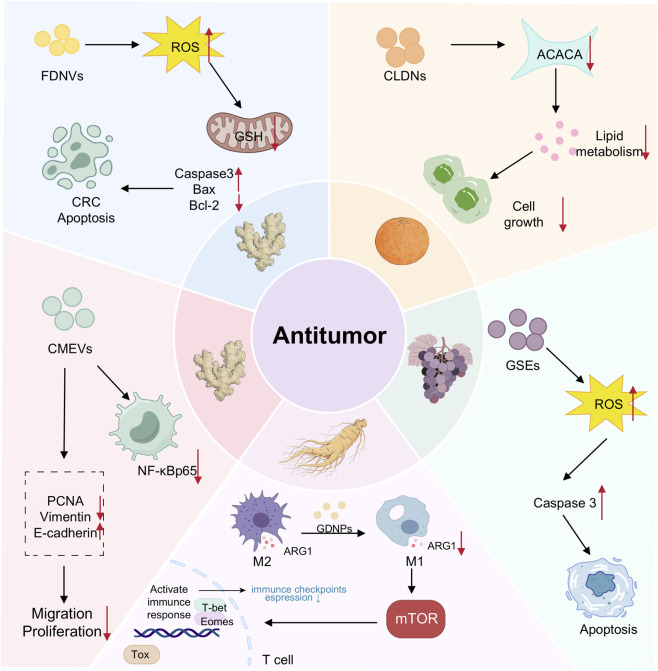
Anti-tumor mechanism. PELNs can directly target colorectal cancer cells, disrupt the intracellular redox homeostasis, promote cancer cell apoptosis, and impair their invasive capacity. Additionally, PELNs can modulate the tumor microenvironment and reprogram tumor-associated macrophages (TAMs) to alleviate colorectal cancer progression.

Studies have shown that orally administered PELNs offer unique advantages in the treatment of intestinal tumors (Figure 5). PELNs can withstand the harsh conditions of the gastrointestinal tract, protecting their encapsulated active ingredients from degradation (Yi et al., 2024). They ultimately deliver these ingredients precisely to colon tumor sites (Chen et al., 2023). Once they reach the target site, they can release their encapsulated active ingredients via mechanisms such as membrane fusion or endocytosis.

Ginger-derived nanovesicles (GDNVs) can be specifically internalized by colorectal cancer cells, disrupt the intracellular redox homeostasis, compromise the tumor cell survival environment, and ultimately induce apoptosis in colorectal cancer cells (Wongkaewkhiaw et al., 2022). Curcumin-loaded exosomes can suppress NF-κB p65 expression, thereby inhibiting the proliferation and migration of colorectal cancer cells and exerting anti-tumor effects (Xu and Liu, 2024). Exosome-like particles isolated from lemon juice can downregulate the expression of acetyl CoA carboxylase 1 (ACACA), reduce lipid metabolism, and inhibit the phosphorylation of extracellular signal-regulated kinase 1/2 and p38 mitogen-activated protein kinase, thereby suppressing colorectal cancer cell proliferation without affecting normal cells (Raimondo et al., 2018). Grape-derived exosomes are rich in anthocyanins, which reduce mitochondrial membrane potential, activate the caspase-dependent apoptotic pathway, and inhibit colon cancer cell proliferation (Quero et al., 2021).

PELNs can also suppress colorectal cancer by remodeling the tumor microenvironment (TME). The TME is a highly dynamic and interactive ecosystem composed of cancer cells, fibroblasts, immune cells, vascular endothelial cells, neurons, and the extracellular matrix (ECM) (Fatima, 2025). In colorectal cancer, chronic inflammation, microbial dysbiosis, and metabolic reprogramming are the key factors driving the remodeling of the TME (Song J. et al., 2025). Elevated levels of pro-inflammatory cytokines (e.g., IL-6, TNF-α) and immunosuppressive factors (e.g., TGF-β, IL-10) promote cancer cell proliferation, angiogenesis, and immune evasion (Nakase and Shimomori, 2025; Podlesnaya et al., 2022). Ginseng-derived nanoparticles (GDNPs) reprogram tumor-associated macrophages (TAMs) through the mTOR-T-bet axis, modulate the release of ARG1, and subsequently alleviate T cell exhaustion within the TME. Interestingly, the authors also observed that GDNPs significantly ameliorated symptoms of colorectal cancer in MC38 tumor-bearing mice (Lv et al., 2023).

### 5.4 Modulation of the gut microbiota

Intestinal dysbiosis serves as a critical pathological basis for various gastrointestinal disorders, attributable to multiple factors including diet, environmental exposures, and host genetics. Dysbiosis is characterized by a reduction in obligate anaerobic bacteria, an increased proportion of opportunistic pathogens, including *Staphylococcus aureus*, decreased synthesis of short-chain fatty acids, and accelerated pathogen evolution (Blumberg and Powrie, 2012; Pham and Lawley, 2014). An imbalanced gut microbiota may lead to immune dysfunction, disruption of the intestinal barrier, and metabolic disturbances, contributing to the development of various diseases such as IBD, non-alcoholic fatty liver disease, and colorectal cancer (Abreo Medina et al., 2025). Furthermore, studies indicate that gut dysbiosis can influence the central nervous system via the gut-brain axis, potentially contributing to the onset of neurodegenerative disorders (Wachamo and Gaultier, 2025). Hence, strategies aimed at modulating the gut microbiota are of paramount importance for both the treatment and prevention of intestinal-related diseases.

**FIGURE 6:**
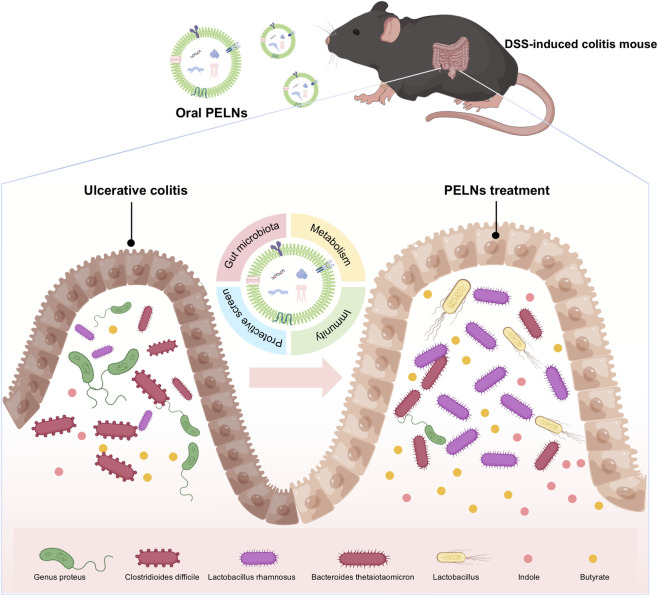
Reconstructing the gut microbiota. PELNs can reduce the proportions of Proteobacteria and Clostridioides difficile in the intestine, while promoting the growth of *Lactobacillus* rhamnosus and Firmicutes (etc.). Meanwhile, PELNs alter the metabolic profile, facilitate the production of indole, acetic acid, and butyric acid, regulate bile acid homeostasis, and thereby maintain immune homeostasis.

PELNs exhibit significant potential in modulating the gut microbiota (Figure 6). Studies indicate that certain PELNs can promote the growth of beneficial bacteria and inhibit the proliferation of pathogenic bacteria, thus regulating the diversity and overall abundance of the intestinal microbiota (Zu et al., 2021; Qiu et al., 2023).

For example, following oral administration of phospholipid acid (PA)-rich ginger-derived exosome-like nanoparticles (GELNs), *Lactobacillus rhamnosus* GG (LGG) preferentially takes up and processes these vesicles in the gut. The metabolites released by LGG subsequently modulate the growth of other intestinal bacterial populations. Furthermore, GELNs contain various microRNAs targeting LGG, including mdo-miR7267–3p, which further induces the production of IL-22, thereby ameliorating murine colitis (Teng et al., 2018).

In another study, garlic-derived exosome-like nanoparticles (GELNs) effectively alleviated inflammatory symptoms in a mouse model of colitis; notably, the GELNs-enriched peu-miR2916-p3 significantly promoted the growth of *Bacteroides* thetaiotaomicron (Wang X. et al., 2024) Additionally, oral administration of folium artemisiae argyi-derived exosome-like nanovesicles (FAELNs) resolved gut microbial dysbiosis in mice with DSS-induced colitis, involving changes in bacterial genera such as *Campylobacter*, *Bacteroides*, Desulfovibrio, and *Helicobacter* (Li Y. et al., 2025).

Moreover, PELNs can further promote the health of the gut microbiota through mechanisms such as enhancing intestinal barrier function, modulating immune responses, and influencing metabolism. For instance, garlic-derived exosome-like nanoparticles have been demonstrated to improve intestinal barrier integrity, restore the composition of the gut microbiota, and reduce intestinal inflammation (Zhu Z. et al., 2023).

Atractylodes macrocephala-derived extracellular vesicle-like particles (AMEVLPs) promoted increased alpha diversity of the gut microbiota and the restoration of beneficial microbial communities. They also altered tryptophan metabolism, resulting in elevated indole derivatives in the intestine, which contributed to the protection of the intestinal barrier and exerted anti-inflammatory effects (Tan et al., 2025). Houttuynia cordata-derived exosome-like nanoparticles (HELNs) balanced the gut microbiota in mice with colitis and reduced the abundance of harmful bacteria. They targeted specifically the inflamed colon tissues, modulated the immune environment, and attenuated inflammation (Li J. H. et al., 2024). Further studies indicate that elevated levels of butyrate, a primary energy source for intestinal cells, can exert positive feedback to regulate the gut microbiota and barrier function, establishing a beneficial cycle (Zheng et al., 2017). Honeysuckle-derived nanovesicles (HNVs) not only increase the abundance of beneficial bacteria such as Firmicutes and decrease the proportion of pathogenic bacteria like Proteobacteria, thereby reversing dysbiosis, but also alter the metabolic profile by promoting the production of acetate and butyrate and modulating bile acid balance; they also maintain immune homeostasis. Through the “microbiota-metabolite-immune-barrier” regulatory loop, HNVs synergistically promote gut microbiota health (Wang et al., 2025).

These application examples demonstrate the potential of PELNs to modulate gut microbiota and improve intestinal health. They also lay the foundation for future clinical applications. As research on PELNs deepens, their prospects for application in treating gastrointestinal diseases are expected to expand.

In summary, the therapeutic mechanism of PELNs in intestinal diseases does not operate via a single-target mechanism, but rather functions as a synergistic network involving multiple interconnected processes, including immune modulation, barrier repair, and microbiota remodeling. Within this network, several signaling pathways play coordinating roles across different processes. For example, the NF-κB pathway serves as a central switch in immune regulation, controlling M1/M2 macrophage polarization and the release of pro-inflammatory cytokines, while also mediating colorectal cancer cell proliferation and migration in the anti-tumor context, reflecting the mechanistic link between inflammation and tumorigenesis. Furthermore, microbiota-derived metabolites such as short-chain fatty acids (SCFAs) act as key messengers linking microbiota remodeling to other processes. Specifically, they activate anti-inflammatory signals and promote macrophage polarization toward the M2 phenotype; simultaneously, they provide energy to intestinal epithelial cells, enhance tight junction protein expression, and reinforce barrier function, thereby forming a “microbiota-metabolism-immunity-barrier” multidirectional regulatory network. Notably, although plant-derived exosome-like nanovesicles from different sources exhibit variations in their active components and functions, their core therapeutic mechanisms share substantial overlap. Future studies should further elucidate the precise interactions among these mechanisms and explore PELNs-based combination therapies to achieve targeted and synergistic treatment of intestinal diseases.

## 6 *In vivo* fate of PELNs

Currently, most studies investigating PELNs for the treatment of intestinal diseases remain at the proof-of-concept stage, primarily exploring therapeutic mechanisms. In *in vivo* experiments, researchers typically employ doses of 20–100 mg/kg for efficacy evaluation. Notably, some investigators also adopt particle concentration as a dosing parameter, which poses challenges for cross-study comparisons.

Notably, PELNs have exhibited precise targeting toward diseased tissues. In mice with colitis, purslane-derived exosomes exhibited significant colonic enrichment at 6 h post-oral administration, a phenomenon that gradually subsided over 24 h. Interestingly, negligible fluorescence signals were detected in the colon of healthy mice as well as in major organs such as the heart, liver, spleen, lungs, and kidneys (Zhu M. Z. et al., 2023). Centella asiatica-derived exosomes displayed even faster targeting kinetics, reaching peak concentrations in diseased tissues within 2 h after oral administration (Shi et al., 2025).

However, significant gaps persist in the pharmacokinetic data of PELNs. To date, core parameters such as elimination half-life (t_1/2_), peak concentration (C_max_), bioavailability (F), and volume of distribution (Vd) have not been reported. This lack of data severely hinders the evaluation of their clinical translation potential (Feng et al., 2023).

Furthermore, biosafety is an indispensable aspect of evaluating PELNs as candidate drugs. For instance, mice fed coix lacryma-jobi seeds-derived exosome-like nanovesicles (CLS-Exos) for 7 days exhibited no significant organ damage (Xiao L. et al., 2025). Similarly, mice treated with zanthoxylum bungeanum-derived exosome-like nanovesicles (ZbELNs) for 7 days showed normal levels of liver function, kidney function, and myocardial enzymes (Gong et al., 2025). However, these studies focus solely on short-term biosafety validation. Comprehensive data on the long-term safety of PELNs, including assessments of chronic toxicity and long-term immunogenicity, are currently lacking. Addressing these gaps is essential prior to advancing PELNs toward clinical translation.

## 7 Summary and future perspectives

PELNs are considered promising carriers in treating IBD and other gastrointestinal disorders because of their inherent edibility, low immunogenicity, and excellent stability in the gastrointestinal environment. Research shows that PELNs can maintain nanoparticle size and membrane integrity under conditions such as simulated gastric fluid (pH ≈ 2) and pancreatic enzymes, resisting the dual attack of acidity and enzymatic digestion (Song et al., 2025c). This stability enables them to resist acidic and enzymatic challenges and effectively deliver drugs and active ingredients to gastrointestinal sites (Nemidkanam and Chaichanawongsaroj, 2022).

Scientists have confirmed that nanovesicles derived from plants such as ginger, grapefruit, and citrus are rich in bioactive components, including endogenous miRNAs, lipids, and proteins. These nanoparticles possess immunomodulatory activities, antagonize pro-inflammatory factors, and promote gastrointestinal epithelial repair. They can suppress the production of pro-inflammatory cytokines, such as TNF-α and IL-6, while elevating levels of IL-10 and IL-22, significantly alleviating symptoms in DSS-induced colitis models. PELNs can also modulate the gut microbiota, suppress the proliferation of harmful bacteria, restore intestinal barrier function, and mitigate the initiation and progression of intestinal tumors. Furthermore, as a natural drug delivery platform, PELNs can be efficiently loaded with small-molecule drugs or miRNA, through methods such as co-incubation, sonication, or electroporation. Their enhanced release rate in acidic environments further aligns with therapeutic needs in low-pH regions of the intestine.

Currently, liposomes represent the most clinically established class of synthetic nanocarriers and have been widely utilized for therapeutic delivery, as evidenced by multiple FDA-approved formulations that validate their safety and efficacy (Zhang et al., 2026; Filipczak et al., 2020). However, in the context of oral delivery for intestinal diseases, PELNs exhibit several distinct advantages over conventional liposomes. First, liposomes serve primarily as inert delivery carriers lacking intrinsic therapeutic properties. In contrast, PELNs are natural nanovesicles that encapsulate intrinsic bioactive molecules, which may independently mediate therapeutic effects. Second, conventional liposomes are limited by poor stability, rapid degradation, and rapid *in vivo* clearance, all of which hinder their clinical application (Zhu J. et al., 2025). In contrast, PELNs maintain structural stability across a wide pH range, rendering them particularly well-suited for oral delivery targeting intestinal diseases (Zhao et al., 2024). A previous study reported that plant-derived nanocarriers exhibited an internalization rate of more than 80% in A549 cells at 37 °C, which was significantly higher than that of cationic liposomes (approximately 40%), demonstrating the superior cellular uptake efficiency of these plant-derived nanocarriers (Wang et al., 2013). Finally, cationic liposomes are linked to potential toxicity and can readily trigger immune responses (Long et al., 2025). Notably, PELNs exhibit low immunogenicity and high biocompatibility, rendering them promising candidates as drug delivery systems (Langellotto et al., 2024). However, the oral administration of PELNs for treating intestinal diseases also faces several challenges. Currently, there is a lack of unified standards for their extraction, purification, and characterization, which complicates large-scale production (Xiao X. et al., 2025).

In addition, several critical issues warrant more rigorous evaluation, including the *in vivo* metabolic fate of PELNs, their potential immunogenicity, and batch-to-batch variability. To address these concerns, long-term toxicity studies lasting at least 6 months should be conducted to assess both the chronic toxicity and immunogenicity of PELNs in appropriate animal models. In light of these challenges, scientists should focus on developing a diverse range of novel plant-derived, exosome-like vesicles, advancing more precise and efficient engineering technologies, promoting the translation from preclinical studies to clinical trials, and exploring the pivotal roles of PELNs in systemic diseases mediated by the “gut brain axis” and “gut liver axis.

Finally, although the results from animal models are encouraging, human clinical data regarding the use of PELNs for the treatment of intestinal diseases remain scarce. Encouragingly, preliminary clinical trials have been completed and demonstrate significant potential. The most direct evidence comes from a clinical trial completed in the United States, in which ginger-derived exosomes were used to treat IBD, preliminarily validating their safety and tolerability in humans (Author anonymous, 2018) (https://clinicaltrials.gov/study/NCT04879810). In another study, oral administration of atractylodes macrocephala-derived exosome-like vesicle-like nanoparticles (AMEVLPs) to two patients with UC resulted in a reduction of rectal mucosal erosion and purulent secretions compared to baseline. However, this clinical trial was limited by a small sample size, and the findings regarding its efficacy warrant validation through large-scale clinical trials (Tan et al., 2025). Therefore, it is reasonable to anticipate that, through the implementation of more rigorously designed clinical trials, PELNs, as a natural, sustainable, and biocompatible oral delivery system, hold the potential to bridge the current translational gap in the field of plant-derived nanomedicines for intestinal diseases, and to provide new strategies and hope for the clinical treatment of chronic intestinal diseases such as inflammatory bowel disease.

In conclusion, orally administered PELNs hold significant potential for the treatment of intestinal diseases; however, their clinical application continues to face numerous scientific and technological challenges. Through more in-depth fundamental research and rigorous clinical validation, PELNs are anticipated to emerge as a novel, safe, and effective therapeutic approach for patients with gastrointestinal disorders.
